# Conformational diversity in poly‐HAMP arrays and its implications for signal transduction

**DOI:** 10.1002/pro.70743

**Published:** 2026-08-06

**Authors:** Murray Coles, Carolin P. Ewers, Reinhard Albrecht, Mikel Martinez‐Goikoetxea, Malgorzata Orlowska, Jörg Martin, Andrei N. Lupas, Marcus D. Hartmann, Stanislaw Dunin‐Horkawicz

**Affiliations:** ^1^ Department of Protein Evolution Max Planck Institute for Biology Tübingen Tübingen Germany; ^2^ Institute of Evolutionary Biology, Faculty of Biology, Biological and Chemical Research Centre University of Warsaw Warsaw Poland; ^3^ Interfaculty Institute of Biochemistry University of Tübingen Tübingen Germany

**Keywords:** AlphaFold2, class III histidine kinases, coiled coils, conformational switching, HAMP domain, poly‐HAMP arrays, signal transduction, x‐ray crystallography

## Abstract

Prokaryotic transmembrane receptors are built around a helical coiled‐coil backbone, with sensory, modulatory, and effector domains arranged along its length. The modulatory HAMP domain forms a parallel four‐helix coiled coil integrated into this backbone, typically connecting transmembrane segments with downstream cytosolic domains. In many systems, HAMP domains transduce signals through axial rotation of their helices; however, it is not clear how broadly applicable this mechanism is. Here, we describe two families of soluble chemoreceptors and sensory kinases that contain long arrays of concatenated HAMP domains, which we term poly‐HAMP. Although these poly‐HAMP arrays evolved independently, both families share sequence features consistent with convergence on a similar functional system. We determined the crystal structures of 4‐HAMP and 6‐HAMP segments from the poly‐HAMP array of histidine kinase AskA of *Myxococcus xanthus*, revealing unusually close packing between adjacent domains and conformational patterns compatible with the rotational signaling model. To define the broader conformational landscape, we computed AlphaFold2 models for over 200 chemoreceptor‐ and kinase‐associated arrays. The models were consistent with the AskA structures, yet revealed distinct preferences: chemoreceptor arrays were predicted to adopt stable conformations, whereas kinase arrays more frequently adopted potentially less favorable conformations. When modeled in isolation from neighboring domains, HAMP domains from kinase arrays adopted alternative conformations related to their array‐embedded forms by axial helix rotation. Taken together, our results suggest that, despite their independent origins, HAMP‐containing systems including poly‐HAMP arrays and canonical single‐HAMP receptors may share a conserved mode of conformational plasticity involving axial helix rotation.

## 1 INTRODUCTION

Coiled coils are structural protein motifs formed by two or more *α*‐helices that wrap around each other in parallel or antiparallel orientation, forming regular bundles (Lupas et al., 2017; Woolfson, 2023). Initially identified in structural proteins such as keratins, where they provide mechanical stability, coiled coils were later found to serve many functions beyond structural support (Hartmann, 2017; Truebestein & Leonard, 2016). This functional versatility is well illustrated by prokaryotic signaling proteins such as sensor histidine kinases and chemoreceptors, which are built around coiled‐coil backbones with functional domains arranged along them (Diensthuber et al., 2013; Ferris, Coles, et al., 2014; Ferris, Zeth, et al., 2014). In these proteins, the coiled‐coil backbone not only acts as a structural scaffold but also plays a central role in signal transduction, with some functional domains consisting entirely of coiled coils. Among these coiled‐coil‐based domains, one of the best‐studied is the HAMP domain, named after its original identification in histidine kinases, adenylate cyclases, methyl‐accepting proteins, and phosphatases. HAMP domains occur in a broader range of signaling contexts, where they form parallel four‐helix coiled‐coil bundles and function as signal modulators and connectors between domains of different types (Dunin‐Horkawicz & Lupas, 2010; Hulko et al., 2006; Parkinson, 2010).

**FIGURE 1:**
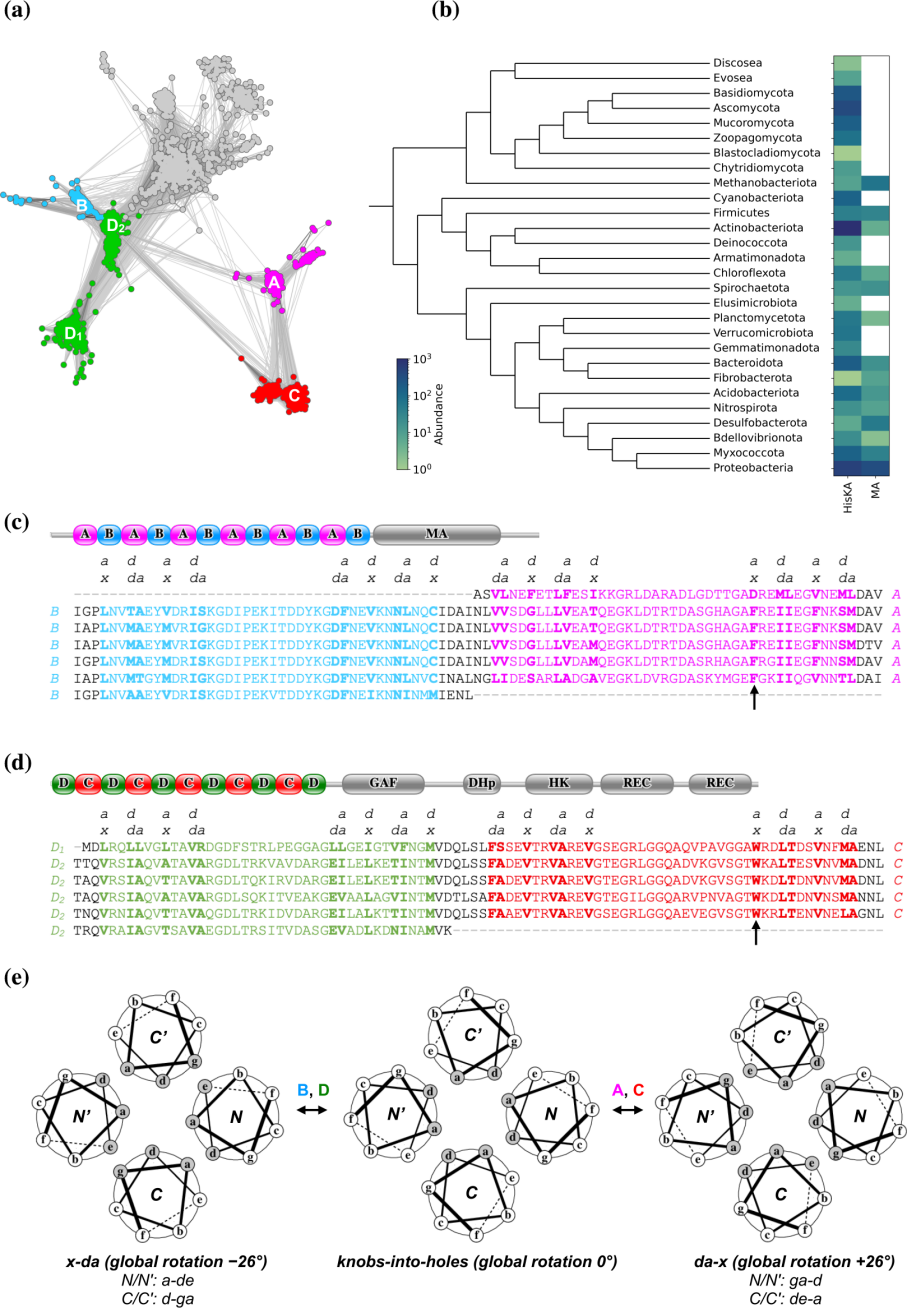
Sequence analysis of poly‐HAMP array. (a) Cluster map of individual HAMP domain sequences, shown as circles positioned according to their pairwise BLAST similarity. Domains from poly‐HAMP arrays are colored according to type (labeled A to D), while domains from single‐HAMP receptors are shown in gray. (b) Phylogenetic distribution of phyla encoding either type of poly‐HAMP arrays. (c) Alignment of di‐HAMP modules from an exemplary chemoreceptor‐associated array (WP_274427672), with canonical and non‐canonical HAMP domains colored in blue and purple, respectively. For each HAMP type, the knobs‐into‐holes and rotated (x‐da or da‐x) registers are indicated above. Residues contributing to the hydrophobic core are shown in bold. The downstream methyl‐accepting (MA) domain is indicated. (d) Equivalent depiction for a kinase‐associated array (AEW99355), with canonical and non‐canonical HAMP domains shown in green and red, respectively. The sensory GAF domain, kinase domains (DHp and CA), and receiver (REC) domains are indicated. (e) Helical‐wheel representations of the idealized x‐da, knobs‐into‐holes, and da‐x packing states of a HAMP domain. N‐ and C‐terminal helices from the two monomers are labeled N/N′ and C/C′, respectively. The heptad registers shown in the wheel diagrams are mapped onto the corresponding x‐da and da‐x packing modes, and the idealized global rotational states are indicated below each arrangement. The proposed conformational ranges of canonical HAMP groups B and D and non‐canonical HAMP groups A and C are indicated between the packing states.

At the sequence level, many coiled coils, including HAMP domains, are characterized by a repeating heptad pattern of seven residues, although other repeat lengths, such as 11 or 15 residues, are also observed in coiled‐coil structures (Lupas & Gruber, 2005; Szczepaniak et al., 2021). The positions within the canonical 7‐residue repeat are labeled *a* through *g*, with positions *a* and *d* typically occupied by hydrophobic residues facing the core of the bundle. These two core residues interact via knobs‐into‐holes packing, in which a residue from one helix (the knob) fits into a cavity formed by the residues of the opposing helices (the hole) (Crick, 1953). Unexpectedly, the first structure of a HAMP domain from the *Archaeoglobus fulgidus* receptor Af1503 revealed a conformation in which all helices were axially rotated by approximately 26° relative to the canonical knobs‐into‐holes packing register (Hulko et al., 2006). In this rotated state, a third residue is incorporated into the hydrophobic core, resulting in a conformation known as x‐da packing (Figure 1e). While this extended packing is common in antiparallel coiled coils and is achieved by axial rotation of all helices in the same direction (Szczepaniak et al., 2014), in a parallel HAMP structure, it requires the input (N‐terminal) and output (C‐terminal) helices to rotate in opposite directions. This observation led to a signaling mechanism hypothesis known as the “gearbox” model, in which HAMP interconverts between the knobs‐into‐holes and x‐da states (Figure 1e) (Hulko et al., 2006). This “gearbox” model is consistent with findings from structural and functional studies in diverse HAMP‐containing systems (Airola, Watts, Bilwes, & Crane, 2010; Ferris et al., 2011, 2012; Ferris, Zeth, et al., 2014; Hulko et al., 2006; Klose et al., 2014; Mondéjar et al., 2012). A complementary view is provided by the dynamic‐bundle model, which relates signaling output to the packing stability and dynamics of the HAMP domain. In this model, HAMP is proposed to sample a range from tightly packed to highly destabilized states, with kinase activity depending biphasically on bundle stability, as supported primarily by studies of the Tsr chemoreceptor system (Parkinson, 2010; Stewart, 2014; Sukomon et al., 2017; Zhou et al., 2011). Because these states are defined by bundle stability and dynamics rather than by a particular core‐packing register, they cannot be assigned universally to the knobs‐into‐holes, x‐da, and da‐x nomenclature used in this work, although structural transitions involving helix rotation can be accompanied by changes in conformational dynamics (Airola et al., 2013; Klose et al., 2014).

While most sensor kinases and chemoreceptors contain a single HAMP domain, typically connecting the membrane‐anchoring region to downstream effector domains, some contain multiple copies. In these proteins, HAMP domains are either interspersed with other domains or arranged in poly‐HAMP arrays consisting of many connected HAMP units. Sequence analyses have shown that poly‐HAMP arrays evolved through the repetition of di‐HAMP modules, each composed of two types of HAMP domains: canonical ones, resembling the Af1503 HAMP, and non‐canonical (or divergent) ones, which adopt a similar structure but share little sequence similarity with canonical domains (Airola, Watts, & Crane, 2010; Dunin‐Horkawicz & Lupas, 2010). Both canonical and non‐canonical HAMP sequences exhibit features compatible with knobs‐into‐holes packing, though they differ in the nature of their alternative rotated states. We have previously proposed that while canonical HAMPs transition between knobs‐into‐holes and x‐da states, non‐canonical ones alternate between knobs‐into‐holes and da‐x packing (Figure 1e) (Winski et al., 2024). Both the x‐da and da‐x states incorporate a third residue into the hydrophobic core but result from opposite rotations of the input and output helices. In canonical HAMP domains, the transition to x‐da packing involves counterclockwise rotation of the input helices and clockwise rotation of the output helices. In contrast, in non‐canonical HAMPs, da‐x packing arises from the reverse: clockwise rotation of the input helices and counterclockwise rotation of the output helices. Therefore, placing the two HAMP types in tandem creates a self‐contained toggle: the first reverses the sense of rotation, and the second reverses it again before passing the signal to the next di‐HAMP module. This configuration allows the formation of extended HAMP arrays within a single protein, which may otherwise stall if composed of only one HAMP type.

Poly‐HAMP arrays should be distinguished from a related class of architectures that we refer to here as multi‐HAMP proteins. Unlike poly‐HAMP arrays, which can comprise dozens of HAMP domains arranged in repeating, highly similar, and tightly connected di‐HAMP modules, multi‐HAMP proteins have fewer and more diverse HAMP domains. These domains can be separated by linkers of variable length, ranging from short helices to entire domains such as PAS. A well‐studied example of a multi‐HAMP protein is the soluble receptor Aer2 (Airola, Watts, Bilwes, & Crane, 2010; Anaya et al., 2022), which contains five HAMP domains: three located at the N‐terminus, followed by an oxygen‐sensing PAS domain, and two additional HAMP domains. Another example is HtrII, a two‐HAMP membrane protein that forms a light‐sensing complex with sensory rhodopsin II (Wang et al., 2012).

Although the overall domain organization of poly‐HAMP arrays is known, their biological roles and detailed structural properties remain largely uncharacterized. In bacteria, functional data mainly come from poly‐HAMP histidine kinases of *Myxococcus xanthus*, which have been implicated in developmental regulation and sensitivity to certain fungicides (Ewers, 2015; Shi et al., 2008). In contrast, fungal examples include group III histidine kinases, in which mutations or deletions within poly‐HAMP and other regions affect osmotic stress responses and fungicide sensitivity or resistance (Avenot et al., 2005; Dry et al., 2004; Ma et al., 2006; Yoshimi et al., 2004). Functional studies further indicate that the poly‐HAMP region contributes to osmosensing and fungicide response, placing these kinases upstream of the HOG pathway and linking them to the action of agriculturally important dicarboximide and phenylpyrrole fungicides (Buschart et al., 2012; El‐Mowafy et al., 2013; Kaur et al., 2014; Lawry et al., 2017; Meena et al., 2010; Randhawa et al., 2016). Such kinases have also been linked to virulence in *Botrytis cinerea*, underscoring the broader biological significance of poly‐HAMP arrays (Viaud et al., 2006). This functional diversity prompted us to investigate whether the conformational plasticity of poly‐HAMP arrays reflects a shared signaling mechanism. To address this question, we first performed sequence‐based identification and classification of poly‐HAMP arrays, followed by crystallographic characterization of the first six HAMP domains of the AskA poly‐HAMP array. Given the high accuracy of AlphaFold2 in modeling coiled‐coil domains (Madaj et al., 2025), including HAMP domains in different states (Winski et al., 2024), we then used it to predict the structures of more than 200 poly‐HAMP arrays. By modeling these arrays both as entire assemblies and as individual HAMP domains separated from the array context, we not only extended observations derived from the experimental structures but also identified alternative modeled conformations compatible with the “gearbox model.”

## 2 RESULTS AND DISCUSSION

### 2.1 Identification and classification of poly‐HAMP arrays

We identified poly‐HAMP arrays by scanning the NCBI sequence database with profiles derived from canonical and non‐canonical HAMP sequences from a manually curated set of representative arrays (see Methods). The resulting sequences were combined with HAMP domain sequences from typical single‐HAMP receptors and clustered based on pairwise sequence similarity (Figure 1a). The resulting cluster map revealed four HAMP groups, labeled A through D, that define two distinct types of poly‐HAMP arrays. The first type, consisting of HAMPs A and B, is found upstream of the methyl‐accepting domains characteristic of chemoreceptors. The second type, comprising HAMPs C and D, occurs upstream of multi‐domain modules that include a GAF domain, histidine kinase domains (DHp and catalytic), and one or more receiver domains. The non‐canonical HAMP domains (groups A and C) are distinct from each other and from all other sequences, whereas the canonical ones (groups B and D) are more similar to each other and to HAMPs from single‐HAMP receptors, such as Af1503. Notably, canonical HAMPs in group D can be further subdivided into D1 and D2 subgroups, with D1 HAMPs consistently occupying the first position in the array.

The taxonomic distribution of poly‐HAMP arrays (Figure 1b) reveals their widespread occurrence, while the presence of kinase‐associated arrays in fungi and Amoebozoa may be consistent with a shared ancestral origin of eukaryotic poly‐HAMP histidine kinases (Ewers, 2015; Kabbara et al., 2019). At the domain level, poly‐HAMP arrays consist of repeating di‐HAMP modules that, in many bacterial proteins, are nearly identical within a single array (Figure 1c,d), consistent with expansion by intragenic amplification. Although chemoreceptor‐ and kinase‐associated arrays are composed of clearly distinct HAMP domain types, suggesting independent evolutionary origins, both share strikingly similar features. These include hydrophobicity patterns consistent with knobs‐into‐holes, x‐da, and da‐x conformations, identical inter‐HAMP linker lengths, and conserved glycine residues at HAMP–HAMP junctions, which likely reflect convergence on a shared interdomain packing mode.

### 2.2 Experimental structure of kinase poly‐HAMP

Although poly‐HAMP kinases are broadly distributed (Figure 1b), functional data are available mainly for fungal group III kinases, whereas bacterial systems remain poorly characterized.

**FIGURE 2:**
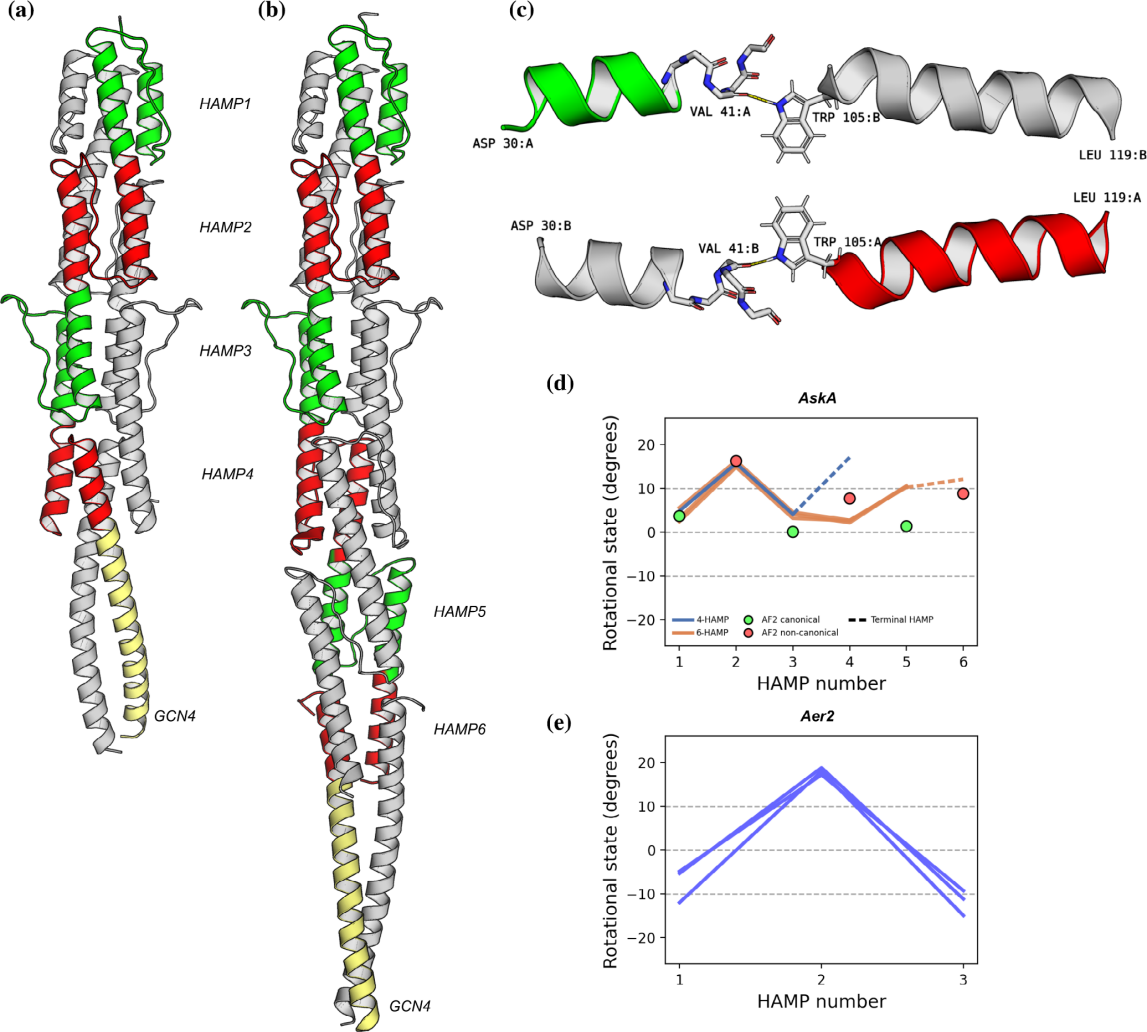
Experimental structures of poly‐HAMP array fragments from the AskA hybrid kinase. Canonical and non‐canonical HAMP domains are shown in green and red, respectively, with the C‐terminal GCN4 stabilizing adaptor in yellow. For clarity, chain A is colored and chain B is shown in gray in panels (a)–(c). (a) Structure of the four‐HAMP construct. (b) Structure of the six‐HAMP construct. (c) Close‐up of the canonical‐to‐non‐canonical interface showing hydrogen bonding between tryptophan 105 side chain and the backbone oxygen of valine 41. For clarity, only N‐terminal and C‐terminal helices of canonical and non‐canonical HAMP, respectively, are shown. (d) Conformations of HAMP domains in the experimental 4‐HAMP and 6‐HAMP structures, shown in blue and orange, respectively, compared with the corresponding domains from the AlphaFold2 model of the full‐length array without GCN4 (colored dots indicate domain type). Dashed line segments indicate the terminal HAMP domains adjacent to the GCN4 adaptor. (e) Conformations of the first three HAMP domains from the Aer2 multi‐HAMP array.

The *M. xanthus* AskA kinase (MXAN_0712; UniProt: Q1DEE3) contains a 21‐HAMP array comprising an N‐terminal D1 HAMP followed by 10 C–D2 repeats, has an overall domain organization similar to that of the better‐characterized fungal group III kinases (Figure 1d), and had previously been associated with deletion phenotypes affecting fruiting‐body formation and sporulation (Ewers, 2015; Shi et al., 2008). We therefore chose AskA to characterize how HAMPs pack and what conformations they assume in a bacterial poly‐HAMP array, and we determined crystal structures of its first four and first six HAMP domains, each fused to a C‐terminal GCN4 adaptor to improve stability (Figure 2a,b). In the crystals of the 6‐HAMP construct, we identified four chains in the asymmetric unit (ASU): two formed a complete dimer within the ASU, whereas the remaining two corresponded to separate monomers that formed dimers only through crystallographic symmetry (Supplementary Figure 1). The three resulting 6‐HAMP dimers were highly similar when compared over HAMP1‐HAMP5, with HAMP6 excluded because of incomplete density: the ASU A/B dimer superposed on the two symmetry‐completed dimers with C*α* RMSDs of 1.22 and 1.20 Å, respectively, whereas the two symmetry‐completed dimers differed by only 0.50 Å. The ASU dimer showed moderate internal asymmetry, with a C*α* RMSD of 2.09 Å between chains A and B. In contrast, the shorter 4‐HAMP construct yielded a single dimer in the ASU. In total, we thus obtained three crystallographically distinct dimers from the six‐domain construct and one from the four‐domain construct.

Inspection of the four‐domain construct revealed that the last HAMP domain deviated significantly from known HAMP structures, displaying an unstructured interhelical linker and distorted coiled‐coil bundle. We suspected that this unusual conformation might be an artifact resulting from the fusion to the GCN4 adaptor. This suspicion was confirmed in the six‐domain construct, where the first five HAMP domains, including the domain that previously appeared partially unstructured and misfolded, adopted their expected conformations, while the final domain exhibited the same deviation as seen in the shorter construct. Given that the first (N‐terminal) HAMP domains in both constructs are highly similar, we concluded that the influence of the GCN4 adaptor is primarily exerted on its adjacent domains. Consequently, for further analysis, we focused on the first three and five HAMPs from the four‐ and six‐domain constructs, respectively.

Both constructs adopt a similar dimeric architecture in which all HAMP domains align along a common two‐fold symmetry axis (Figure 2a,b). Each HAMP monomer consists of a pair of parallel helices connected by a non‐helical segment. This interhelical linker is tightly packed into the interhelical groove through a combination of hydrophobic and polar interactions, and the monomers dimerize to form a four‐helix coiled coil. The third HAMP in the array differs from the others in two respects. First, a proline residue in the N‐terminal input helix induces a characteristic kink. Second, an insertion in the interhelical linker gives rise to a beta‐hairpin structure at its C‐terminal end (Figure 2a,b). These two features are strongly conserved in the third HAMP of AskA‐like poly‐HAMPs, although their functional role remains unclear.

In the context of the array, individual domains are concatenated such that the output C‐terminal helix of each HAMP is continuous with the input N‐terminal helix of the next domain. Likewise, the N‐terminal helix of each HAMP is nearly coaxial with the C‐terminal helix of the preceding HAMP, giving the array the appearance of a continuous, supercoiled four‐helix bundle (Figure 2a–b). In single HAMP domains, each monomer consists of two helices of equal length, offset by one helical turn. As a result, the dimeric unit forms a central four‐helix bundle flanked by short two‐helix segments. In poly‐HAMP arrays, however, these segments overlap completely, bringing neighboring domains into direct contact via backbone hydrogen bonds between the capping motifs of the respective helices (Figure 2c). This close packing provides a rationale for the conservation of glycine residues in these capping motifs (Figure 1c,d). A similarly tight interdomain arrangement has been observed between HAMP2 and HAMP3 in the Aer2 multi‐HAMP module, where Gly82 and Gly139 in the corresponding capping regions participate in analogous structural contacts (Airola, Watts, Bilwes, & Crane, 2010).

The alternating arrangement of canonical and non‐canonical HAMP domains gives rise to two distinct types of interdomain interface. In the canonical‐to‐non‐canonical interface, a conserved tryptophan located at the N‐terminal end of the C‐terminal helix in the non‐canonical HAMP forms a hydrogen bond with the backbone at the C‐terminal end of the N‐terminal helix in the upstream canonical HAMP (Figure 2c). The reverse, non‐canonical‐to‐canonical interface is less specific (Supplementary Figure 2), with another hydrophobic residue lacking hydrogen‐bonding capacity at the corresponding position (Figure 1d). The difference between these two interface types is also reflected in the geometry of the C‐terminal capping motifs of the input helices. Whereas in canonical HAMPs, the N‐terminal helix terminates in a classic six‐residue Schellman motif (Aurora & Rose, 1998), in non‐canonical HAMPs it adopts a less common seven‐residue “wide Schellman” form (Newell, 2025).

The close packing of HAMP domains observed in the experimental structures is reflected by the conservation of glycine residues at HAMP–HAMP junctions in both kinase‐ and chemoreceptor‐associated arrays, as well as by the conservation of tryptophan at the canonical‐to‐non‐canonical interface in kinase arrays (Figure 1d), where it is replaced by a bulky hydrophobic residue, most commonly phenylalanine, in chemoreceptor arrays (Figure 1c). The functional importance of close HAMP–HAMP packing is also reflected in mutagenesis data for fungal poly‐HAMP‐containing group III histidine kinases, where mutations of the conserved glycine residues at HAMP–HAMP junctions are associated with phenotypes as severe as those caused by substitutions affecting hydrophobic core residues (Ewers, 2015; Fillinger et al., 2012; Ma et al., 2006; Yoshimi et al., 2004).

Finally, we analyzed the conformations of individual HAMP domains in the two experimental structures using Crick's parametrization, which enables precise quantification of coiled‐coil structures (Szczepaniak et al., 2021). For HAMP domains, the rotations of the input (N‐terminal) and output (C‐terminal) helices relative to the bundle axis were calculated separately, and the global rotation coefficient was defined as the difference between them (Winski et al., 2024). This coefficient places a given HAMP structure on a continuum of rotational states, with idealized values of approximately −26°, 0°, and +26° corresponding to x‐da, knobs‐into‐holes, and da‐x packing, respectively (Figure 1e). Individual HAMP structures, including mutants, often adopt intermediate values within this continuum (Winski et al., 2024). This approach has the advantage of capturing the global rotational state independently of the degree of bundle supercoiling, allowing fair comparisons across different HAMP types.

Application of this procedure revealed that the structures obtained from the four‐ and six‐domain constructs are highly similar, with only minor differences between corresponding domains. The first and third canonical HAMPs in the array adopt essentially the same knobs‐into‐holes conformation, whereas the intervening non‐canonical HAMP adopts a da‐x‐like packing state (blue and orange lines for the 4‐HAMP and 6‐HAMP constructs, respectively, in Figure 2d). This alternating pattern supports the “gearbox” model, according to which neighboring HAMP domains must operate in distinct conformational ranges. However, the downstream HAMPs adopt unexpected conformations that break this pattern. We suspect that this reflects a gradually increasing influence of the C‐terminal GCN4 fusion along the array: the terminal HAMP domains, indicated by dashed line segments in Figure 2d, are most affected, displaying an unstructured linker and distorted handedness, whereas upstream domains appear structurally intact but are shifted toward unusual rotational states, such that only the first three match the conformations expected within an array. Indeed, the corresponding domains in an AlphaFold2 model of the full‐length array without the GCN4 fusion extend this alternating arrangement through HAMP6 (dots in Figure 2d).

**FIGURE 3:**
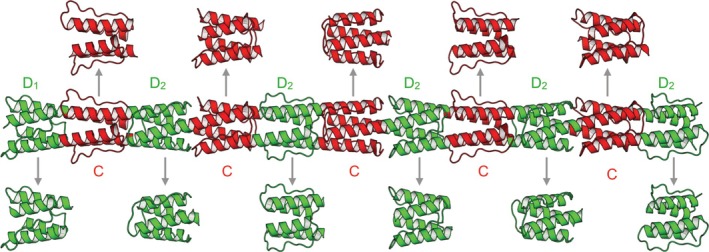
Predicting alternative HAMP conformations. The AlphaFold2 model of the exemplary kinase‐associated poly‐HAMP array (AEW99355) is shown in the center, flanked by models of isolated canonical and non‐canonical HAMP domains below and above, respectively. In all structures, canonical and non‐canonical domains are colored green and red, respectively. As described in the text, HAMP domains modeled in isolation and within arrays adopt distinct rotational states that are related by axial helix rotation, consistent with the gearbox model.

The structure of the AskA array fragment enabled us to describe the atomic details of HAMP–HAMP interactions and revealed evidence for an alternating pattern of conformations. It also expanded the set of canonical HAMP domains known to adopt the knobs‐into‐holes state (previously limited to examples such as CpxA (Mechaly et al., 2014), VicK (Wang et al., 2013), and the Af1503 A291F mutant (Ferris et al., 2011)), as well as the set of non‐canonical domains known to adopt the da‐x conformation which had so far been observed only in the second HAMP domain of the Aer2 multi‐HAMP array structure (Figure 2e) (Airola, Watts, Bilwes, & Crane, 2010). However, because the crystallized fragments covered only part of the AskA array and were locally affected by the C‐terminal GCN4 adaptor, they also raised new questions about whether the observed conformations are generally accessible to both kinase‐ and chemoreceptor‐associated arrays and which conformational rearrangements individual HAMP domains may undergo during signal transduction. To tackle these questions, we modeled over 200 poly‐HAMP arrays and their corresponding isolated HAMP domains with AlphaFold2, following the workflow illustrated in Figure 3, and quantified their rotational states using the same procedure as for the crystallographic models (see Methods). Below, we show that modeling HAMPs both in isolation and in the context of their neighboring domains reveals alternative conformations of canonical and non‐canonical HAMP domains that are compatible with the rotational “gearbox” model.

### 2.3 Conformations of isolated HAMP domains

**FIGURE 4:**
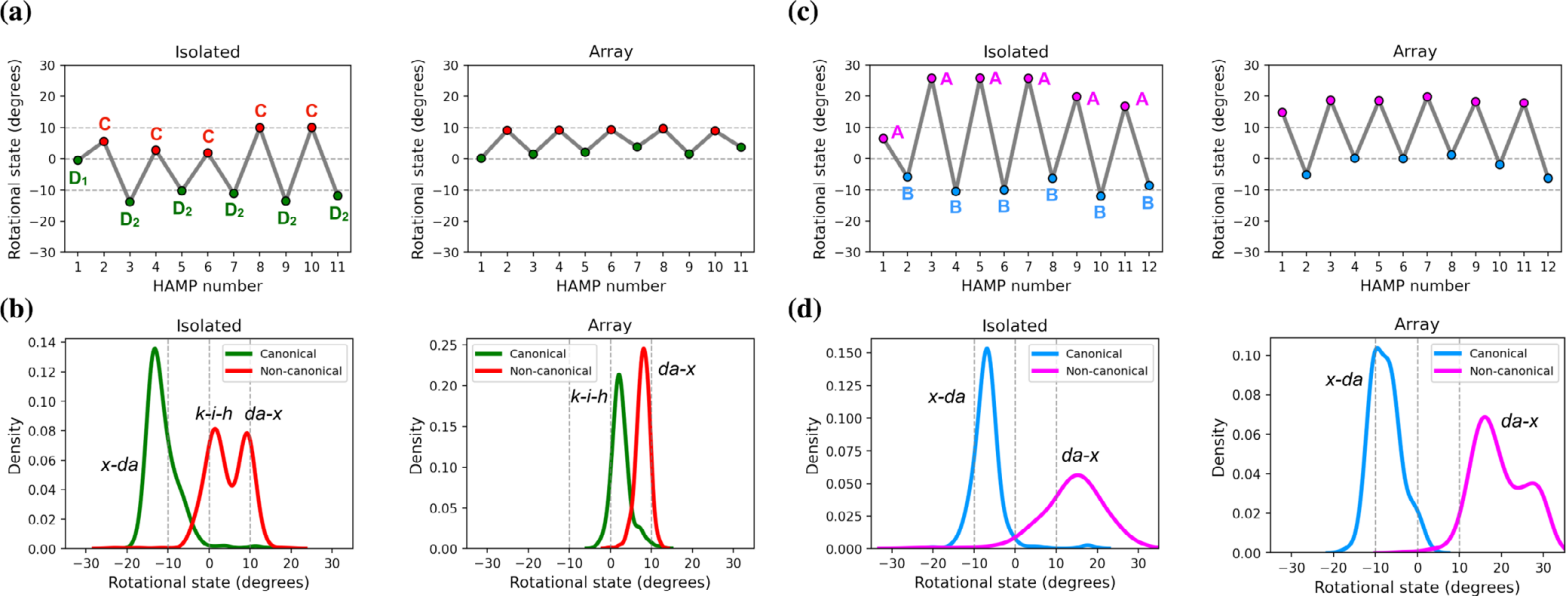
Comparison of isolated and arrayed HAMP conformations. (a) Rotational states of isolated HAMP domains from the kinase‐associated array AEW99355 (left) and of HAMP domains extracted from the complete array model (right). Rotational states corresponding to canonical knobs‐into‐holes packing (k‐i‐h) and rotated x‐da and da‐x packings are indicated. (b) Distribution of rotational states across isolated HAMP domains from 120 kinase‐associated arrays (left) and HAMP domains extracted from complete array models (right). Panels (c) and (d) show corresponding representations for the chemoreceptor‐associated array WP_274427672 and for a representative set of 99 arrays of this type.

In previous work, we showed that AlphaFold2 models of HAMP domains adopt conformations closely matching those observed in corresponding experimental structures (Winski et al., 2024). Since most of these structures were crystallographic, we hypothesize that an isolated HAMP domain modeled in AlphaFold2 without neighboring domains may preferentially adopt a resting‐like conformation. Based on this idea, we generated AlphaFold2 models of 11 individual HAMP domains from AEW99355.1, an exemplary kinase‐associated array shown in Figure 3. These models revealed an alternating pattern of conformations (Figure 4a, left panel), with canonical HAMPs typically adopting the x‐da state and non‐canonical HAMPs adopting either the knobs‐into‐holes state or the da‐x state. The same pattern was observed across isolated HAMP domains obtained from a diverse set of 120 kinase‐associated arrays, where canonical HAMPs consistently adopted an x‐da conformation, while non‐canonical HAMPs sampled two alternative states (knobs‐into‐holes or da‐x; Figure 4b, left panel). Notably, among non‐canonical HAMPs with identical or nearly identical sequences, both the positively rotated da‐x and the knobs‐into‐holes conformations were observed, suggesting that these reflect intrinsic conformational flexibility of individual domains rather than sequence‐determined structural subtypes. A similar alternating pattern was observed for an exemplary chemoreceptor‐associated array (WP_274427672.1; Figure 4c, left panel) as well as in a set of 99 chemoreceptor‐associated arrays (Figure 4d, left panel), where canonical and non‐canonical HAMPs adopt negatively and positively rotated states, respectively. However, these arrays lacked the distinct conformational alternatives observed in non‐canonical HAMPs of kinase arrays.

Modeling of individual HAMP domains highlighted an alternating zig‐zag pattern of resting conformations in both kinase‐ and chemoreceptor‐associated poly‐HAMP arrays (Figure 4). Despite this shared organization, the conformational ranges sampled by canonical and non‐canonical HAMPs differ between the two array types, with both x‐da and da‐x states shifted toward higher rotational values in chemoreceptor‐associated arrays than in kinase arrays. This suggests that, like the characteristic hydrophobicity patterns and conserved HAMP–HAMP linker lengths, the alternating pattern represents a convergent solution that arose independently in kinase and chemoreceptor‐associated arrays to satisfy the constraints of a common conformational principle.

### 2.4 HAMP conformations in poly‐HAMP arrays

According to the gearbox model, canonical and non‐canonical HAMPs in poly‐HAMP arrays should operate within two partially overlapping rotational ranges. Both share the knobs‐into‐holes state but differ in their alternative conformations, adopting x‐da and da‐x packing, respectively. In models of isolated non‐canonical HAMPs from kinase‐associated arrays, we observed the two expected conformations corresponding to the knobs‐into‐holes and da‐x states (Figure 4a,b, left panels). In contrast, canonical HAMPs consistently adopted a single conformation matching the negatively rotated x‐da state, as seen in single‐HAMP receptors such as Af1503. We hypothesized that modeling these HAMPs in the context of their neighboring domains might better capture interdomain interactions and trigger conformations not observed in isolated models (Figure 3). Indeed, modeling 120 full‐length kinase‐associated arrays revealed a marked shift (Figure 4a,b, right panels): canonical HAMPs now adopted the knobs‐into‐holes conformation not seen in isolation, whereas non‐canonical HAMPs that had previously remained in the knobs‐into‐holes state in isolation shifted into the positively rotated da‐x state.

**FIGURE 5:**
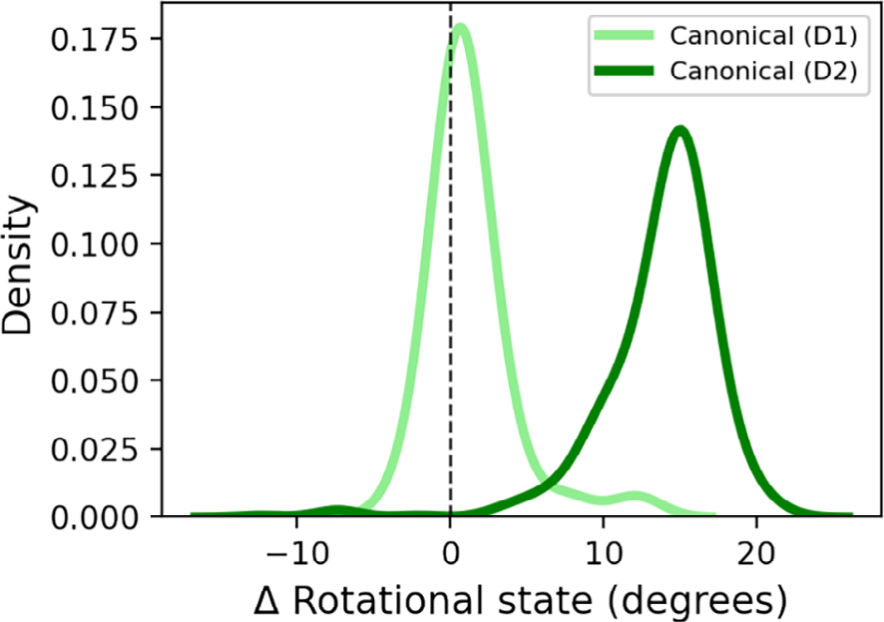
Conformational stability of the first and subsequent canonical HAMPs in kinase‐associated arrays. The plots show the difference in rotational states between isolated and arrayed models for canonical D1 HAMPs (light green) and canonical D2 HAMPs (green). A value of zero indicates that the isolated and arrayed structures adopt the same conformation.

We also noted characteristic behavior of D1‐type HAMP domains, which occur exclusively at the beginning of kinase arrays (Figure 1d). Whereas D2‐type canonical and C‐type non‐canonical HAMPs adopted different conformations in their isolated and array‐embedded forms, D1‐type HAMPs consistently adopted the same conformation regardless of modeling context (Figure 5). We hypothesize that the conformational bias observed in these N‐terminal HAMP domains may reflect a specific role. This possibility is consistent with studies suggesting that individual domains within poly‐HAMP arrays may contribute differently to signaling (Kaur et al., 2014, 2021).

We employed the same procedure to explore possible alternative conformations in 99 chemoreceptor‐associated poly‐HAMP arrays. However, in this case, we observed only modest differences between isolated and array‐embedded models (compare left and right panels in Figure 4c,d), rather than clearly distinct conformational states. Moreover, there was no clear difference between the N‐terminal capping HAMPs that initiate the array and the downstream domains. This further emphasizes that, despite sharing a similar zig‐zag pattern of conformations, kinase‐ and chemoreceptor‐associated arrays may differ in their conformational preferences, with possible implications for signal propagation.

Because these conclusions rely on the top‐ranked AlphaFold2 models, we assessed the robustness of the observed differences between the isolated and arrayed forms by analyzing structural diversity within the full set of 25 ranked predictions (Supplementary Figure 3). For each HAMP domain, dRMSD values for ranks 2–25 relative to rank 1 were compared with the dRMSD between the corresponding isolated and arrayed rank‐1 models, which served as the baseline. For array‐derived HAMP models, 98.8% of rank 2–25 predictions showed lower dRMSD to rank 1 than this baseline, indicating that structural variability among the array‐derived predictions was generally smaller than the difference between the arrayed and isolated forms. For isolated HAMP models, this fraction was 74.3%, indicating greater variability among predictions. However, among the isolated predictions that exceeded the baseline, approximately 45% showed clearly aberrant HAMP geometries and did not form the expected parallel four‐helix bundle. Thus, part of the excess variability among isolated predictions reflects non‐native dimer geometries rather than alternative native‐like HAMP conformations. Importantly, among the rank‐1 models selected for downstream analyses, aberrant geometries occurred in 11.4% of isolated models and 0.8% of array‐derived models, and were excluded during coiled‐coil parameter analysis.

Taken together, these results indicate that the observed differences between the top‐ranked array‐derived and isolated models are unlikely to arise solely from AlphaFold2 uncertainty. Nevertheless, additional native‐like conformations may remain unsampled. This limitation is illustrated by the eighth and tenth C‐type HAMP domains of the AEW99355 array (Figure 4a), which adopt essentially the same conformations in their isolated and arrayed forms. However, when applied across a diverse set of proteins, the current approach collectively captures the conformational states expected from the rotational gearbox model. Finally, we note that, although individual HAMP conformations in full‐array models follow a regular alternating pattern, the particular combination of these states in a given model may not necessarily correspond to a single physiologically accessible state of the entire array.

### 2.5 Implications for signal transduction mechanism

In typical single‐HAMP receptors, membrane‐spanning sensory regions can generate signals such as piston‐like motions (Falke & Hazelbauer, 2001; Gushchin et al., 2017) or axial rotations (Moukhametzianov et al., 2006), which are then received by the HAMP domain and converted into a form interpretable by the downstream, usually effector, domain. In contrast, HAMP domains in poly‐HAMP arrays are positioned downstream of other HAMP domains, and the entire array typically lacks an upstream input domain (Dunin‐Horkawicz & Lupas, 2010). However, a recent study has shown that the N‐terminal, positively charged extension of the *M. xanthus* FrzCD multi‐HAMP chemoreceptor can bind to DNA (Jazleena et al., 2025), suggesting that HAMP arrays may be capable of coupling inputs at their extreme N‐terminus to downstream signaling outputs. DNA association has also been reported for the *M. xanthus* poly‐HAMP histidine kinase AskB, a close paralog of AskA with a similar domain architecture but a longer, 31‐HAMP array (Marcos‐Torres et al., 2020).

Chaining HAMPs into arrays gives rise to a zig‐zag conformational pattern, as seen both in the AskA experimental structure and in modeled arrays (Figures 2 and 4). Despite differences such as variable intra‐HAMP linker lengths and the absence of repetitive di‐HAMP modules, a similar pattern is observed in the structure of the first three HAMP domains of the multi‐HAMP array of the Aer2 soluble chemoreceptor (Airola, Watts, Bilwes, & Crane, 2010) (Figure 2e). The first and third HAMPs adopt negatively rotated states matching the x‐da packing expected for canonical HAMPs, whereas the second adopts the da‐x packing typical of non‐canonical ones. Interestingly, the sequence of the second Aer2 HAMP domain falls within group A HAMPs (Figure 1a), which includes non‐canonical domains of chemoreceptor arrays. This suggests that Aer2‐like multi‐HAMP arrays and the chemoreceptor poly‐HAMP arrays analyzed here may, at least in part, share a common evolutionary origin. An alternating conformational pattern was also proposed for the HtrII two‐HAMP photoreceptor (Wang et al., 2012). Although no experimental structures are available in this case, fluorescein probe accessibility scanning indicated opposite motions in the first and second HAMP domains, consistent with a zig‐zag conformational pattern.

Despite general structural similarities suggesting a shared mechanism of signal transduction, kinase‐ and chemoreceptor‐associated poly‐HAMP arrays exhibit distinct behaviors. In the kinase family, pronounced conformational differences are observed between isolated and array‐embedded forms, whereas in the chemoreceptor family, the two forms are very similar (Figure 4). We hypothesized that the conformational discrepancy in the kinase family may arise from conformational constraints imposed on HAMP domains embedded within an array. These constraints could stem from specific interdomain interactions unique to kinase‐associated arrays, such as the conserved tryptophan‐mediated hydrogen bonds (Figure 2c). In isolated models, where these contacts are absent, HAMP domains may relax into alternative, unconstrained conformations that are otherwise inaccessible within the array.

**FIGURE 6:**
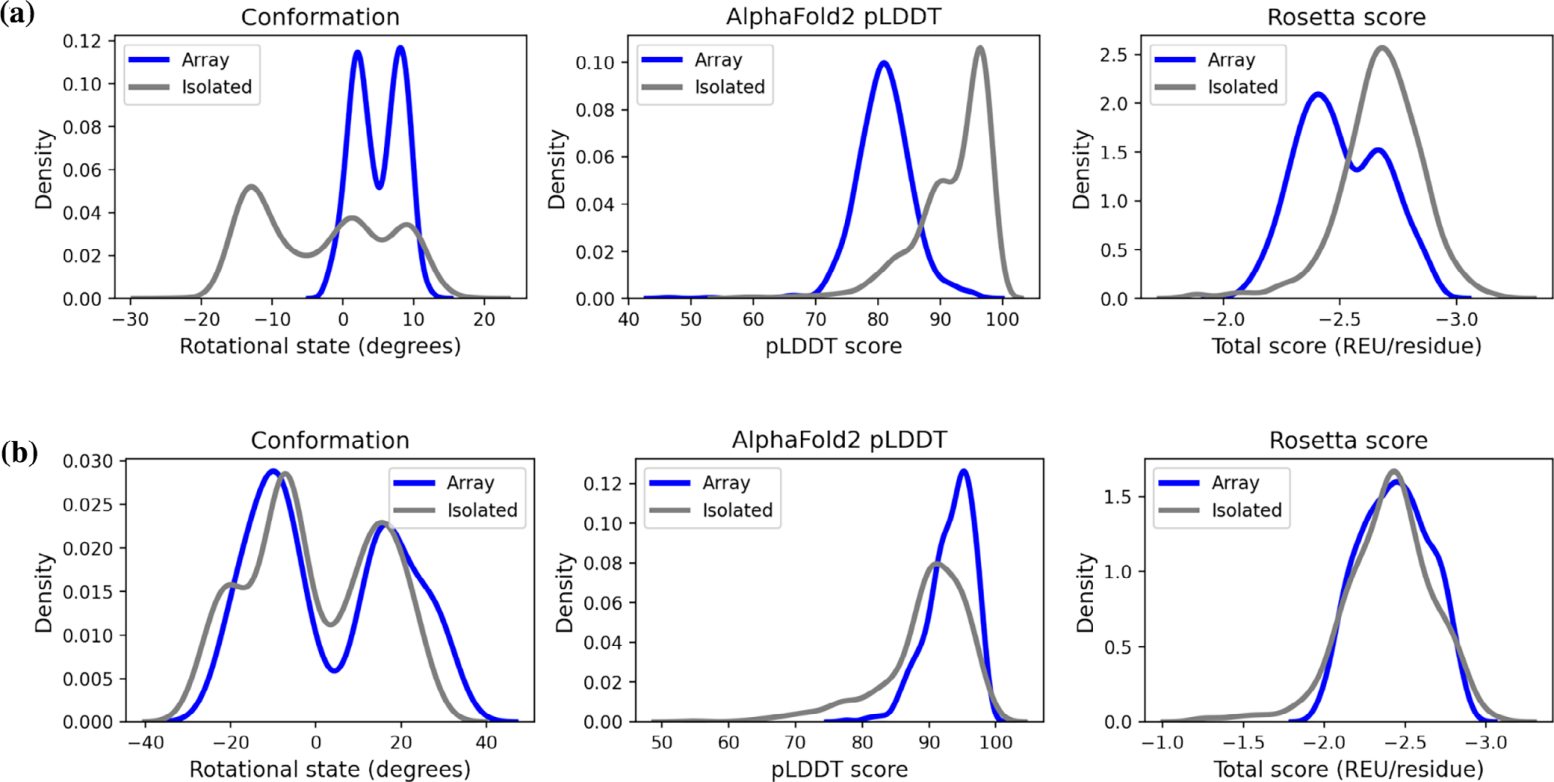
Comparison of AlphaFold2 pLDDT scores and per‐residue Rosetta scores between arrayed and isolated HAMP models. (a) Distributions of conformational states, pLDDT scores, and Rosetta scores for arrayed (blue) and isolated (gray) HAMP domains from kinase‐associated arrays. (b) Same analysis as in (a), shown for chemoreceptor‐associated arrays.

To test this, we analyzed AlphaFold2 pLDDT scores and Rosetta scores for isolated and arrayed domains. While the Rosetta score is often used as a proxy for relative stability (Alford et al., 2017), AlphaFold2's pLDDT score primarily reflects model confidence, but it has also been associated with flexibility (Guo et al., 2022; Ma et al., 2023; Vander Meersche et al., 2025). The results obtained reveal a clear distinction between kinase and chemoreceptor families: in kinase arrays, isolated models exhibit consistently higher pLDDT scores and lower per‐residue Rosetta scores, whereas in chemoreceptor‐associated arrays, the differences are minimal (Figure 6 and Supplementary Figure 4). This suggests that HAMP domains in kinase arrays may populate more dynamic conformations, consistent with the dynamic bundle model, in which the HAMP domain transduces signals by switching between stable and dynamic states (Stewart, 2014; Sukomon et al., 2017; Zhou et al., 2011). A similar mechanism has been proposed for the Aer2 multi‐HAMP receptor, where molecular threading analysis indicated increased conformational freedom of the second, non‐canonical HAMP domain (Airola, Watts, Bilwes, & Crane, 2010).

**FIGURE 7:**
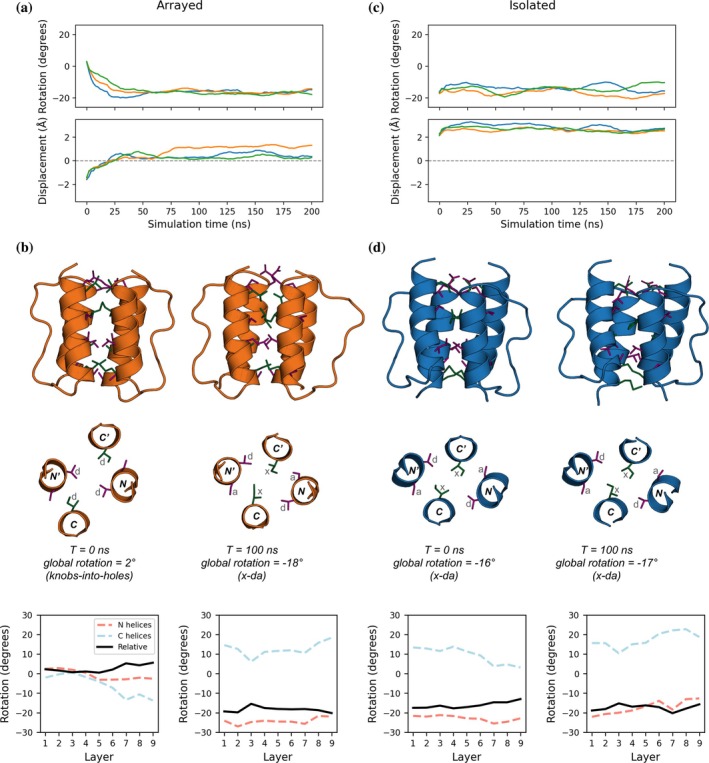
Molecular dynamics simulations of array‐derived and isolated HAMP5 models. (a), (c) Time evolution of global axial helix rotation and cross‐sectional displacement in 250 K simulations of the fifth HAMP domain of the CAA9318664.1 array, corresponding to residues 214–256. Three independent replicates are shown for simulations initiated from the array‐derived model (a) and from the isolated model (c). Cross‐sectional displacement was calculated as the difference between the mean radial distances of two opposite helix pairs; values close to zero indicate a more balanced, near‐square cross‐section, whereas positive and negative values indicate opposite rhomboid‐like arrangements. (b), (d) Representative snapshots from replicate 1 at 0 and 100 ns for the array‐derived (B) and isolated (d) simulations. For each snapshot, the upper cartoon shows the HAMP domain structure, the middle diagram shows the corresponding bundle cross‐section, and the lower plot shows layer‐wise Crick‐angle deviations. Residues at x core positions are shown in dark green, and residues at *a*/*d* core positions are shown in magenta. For the array‐derived 0 ns snapshot, labels indicate the sequence‐based heptad register of the knobs‐into‐holes state; for the remaining snapshots, labels indicate the structural register of x‐da packing. N and C denote the N‐ and C‐terminal HAMP helices of one monomer, whereas N′ and C′ denote the corresponding helices of the second monomer. In the lower plots, dashed red, dashed cyan, and black lines indicate layer‐wise rotations of the N‐terminal helices, C‐terminal helices, and their relative rotation, respectively. The global rotation value is indicated below each cross‐section.

Finally, we used molecular dynamics simulations (MD) of the fifth, canonical HAMP domain from the CAA9318664.1 array to investigate the structural basis of conformational switching. The array‐derived simulations were initiated from a structure extracted from the AlphaFold2 model of the full array. This structure adopted an unrotated knobs‐into‐holes packing that, upon release from the constraints imposed by neighboring domains, rapidly transitioned to a negatively rotated x‐da conformation (Figure 7a). This relaxation involved not only rotation of the helices toward the x‐da state but also a change in the bundle cross‐section from a rhomboid arrangement, in which the input N‐terminal helices are closer to each other, to a more symmetric, near‐square arrangement in which the four helices are approximately equidistant (Figure 7b). Moreover, at later time points in one replicate (shown in orange in Figure 7a), we observed a further change toward the opposite rhomboid arrangement, in which the output C‐terminal helices are closer to each other. In contrast, simulations initiated from the isolated model of the same HAMP remained in the x‐da state throughout the simulations (Figure 7c), while maintaining a relatively stable opposite rhomboid cross‐section in which the output C‐terminal helices were closer to each other (Figure 7d). Thus, relaxation of the array‐derived HAMP model leads to convergence toward the isolated form; however, this convergence is nearly complete for axial rotation but only partial for cross‐sectional shape (Figure 7a,c; Supplementary Figure 5).

Such coordinated changes in axial rotation and cross‐sectional shape are consistent with our previous computational study, in which we showed that family‐wide conformational variability of HAMP domains can be decomposed into differences in axial rotation and cross‐sectional shape, as well as axial shift and helix tilting (Winski et al., 2024). Coupling between HAMP packing transitions and changes in bundle geometry has also been described in other HAMP‐related systems, including experimental structures of Af1503 HAMP mutants (Ferris et al., 2011), MD simulations of NpHtrII (Gushchin et al., 2013) and Af1503 (Zhu et al., 2013) HAMP domains, and structural analyses of the coiled‐coil methyl‐accepting/signaling domain downstream of HAMP in chemoreceptors (Ferris, Zeth, et al., 2014).

Together, these results suggest that the discrete conformations obtained with AlphaFold2 can be connected through continuous transitions in MD, supporting the physical plausibility of interconversion between HAMP conformational states once array constraints are released. Although this analysis was limited to a single domain, it illustrates the potential of molecular dynamics to probe HAMP conformational switching, for example by testing in longer array models whether an imposed rotation propagates between neighboring domains.

As discussed above, fungal group III histidine kinases and the genetically tractable *M. xanthus* AskA system provide full‐length platforms in which the proposed model could be tested using functional readouts appropriate to each system, including osmotic‐stress responses, fungicide sensitivity, and HOG‐pathway activation for fungal kinases, and developmental and sporulation phenotypes in *M. xanthus* (Buschart et al., 2012; El‐Mowafy et al., 2013; Ewers, 2015; Kaur et al., 2014; Randhawa et al., 2016; Shi et al., 2008). Our structures and models provide a framework for extending to poly‐HAMP arrays the mutational approaches used in mono‐ and oligo‐HAMP systems, in which substitutions at selected HAMP core positions have been shown to shift conformational equilibria involving axial helix rotation and to alter signaling output (Airola et al., 2013; Ferris et al., 2011, 2012; Mondéjar et al., 2012). Analogous mutations could be introduced into selected canonical and non‐canonical domains of full‐length poly‐HAMP proteins and tested using the corresponding functional readouts. Particularly informative would be combinations of mutations in different HAMP domains designed to produce compensatory effects, thereby testing conformational coupling within the array.

## 3 CONCLUSIONS

Based on the experimental structure of a poly‐HAMP array and computational analyses, we propose that HAMP domains within poly‐HAMP arrays operate along a conformational continuum ranging from x‐da to knobs‐into‐holes to da‐x packing, with the first and last conformations predominantly accessible to canonical and non‐canonical HAMPs, respectively. The conformational state of an individual HAMP domain depends on its array context (Figure 4), suggesting conformational coupling between neighboring domains, consistent with their tight packing (Figure 2) and the requirement for compatible alternating states within the array (Figure 4). In kinase‐associated arrays, this coupling may additionally involve conformational tension, as suggested by the less favorable states of array‐embedded HAMP domains and their relaxation upon isolation (Figures 6 and 7). Similar concepts have been considered in the context of other two‐component signaling systems, including alternative domain states and allosteric coupling in PhoQ (Mensa et al., 2021), coupled rearrangements of successive signaling modules in phytochrome histidine kinases (Burgie et al., 2024; Wahlgren et al., 2022), and a proposed role for conformational tension in signal transmission through BvgS (Dupré et al., 2013). Given the length of poly‐HAMP arrays, simultaneous conformational switching of all HAMP domains appears unlikely; signal propagation may therefore occur either through waves of local relaxation to alternative states or through shifts in a pre‐existing equilibrium between interconverting packing modes, with the signal stabilizing one state over another.

While some multi‐HAMP arrays, such as that of the HtrII receptor, receive input signals at or near their N‐terminal regions, poly‐HAMP arrays typically lack dedicated upstream sensory domains. One apparent exception is the *M. xanthus* receptor FrzCD, whose N‐terminal positively charged extension binds DNA (Jazleena et al., 2025). The *M. xanthus* poly‐HAMP histidine kinase AskB was also reported to bind DNA in vitro, although the underlying mechanism has not been established (Marcos‐Torres et al., 2020). Therefore, the role of signaling via poly‐HAMP arrays remains unclear, raising the possibility that they act as modulators whose properties can be tuned through evolutionary changes in array length, for example via amplification and loss of di‐HAMP modules. Such changes are reflected both in the high internal similarity between di‐HAMP modules in many arrays (Figure 1c,d) and in examples of related arrays that differ in the number of di‐HAMP modules, patterns that have also been described for some fungal class III kinase arrays (Defosse et al., 2015; Randhawa et al., 2016). Another possibility is that individual HAMP domains may possess intrinsic ligand‐recognition capabilities or undergo post‐translational modifications that influence their conformational landscape. The latter is consistent with studies on fungal group III histidine kinases suggesting that stress‐ or fungicide‐associated chemical modification of residues within HAMP regions may affect signaling (Brandhorst et al., 2019).

## 4 METHODS

### 4.1 Sequence analysis

In 34 chemoreceptor and kinase sequences (Supplementary Table 1), HAMP domains were identified using HH‐suite (Steinegger et al., 2019), pLM‐BLAST (Kaminski et al., 2023), and Foldseek (van Kempen et al., 2024). Identified HAMP sequences were grouped by similarity using CLANS (Frickey & Lupas, 2004), yielding nine clusters. Sequences in each group were aligned with PROMALS3D (Pei et al., 2008), and the resulting multiple sequence alignments were manually curated, including assignment of the coiled‐coil register to the alignment columns. The curated alignments were used to build HMM profiles with HMMER 3.1b2 (Eddy, 2011). These profiles were then used to search the NCBI non‐redundant database clustered at 90% sequence identity (NR90; version dated May 3, 2023) using hmmsearch with default parameters. The resulting matches were concatenated, yielding 5501 proteins (4778 histidine kinases and 723 chemoreceptors) containing poly‐HAMP arrays with five or more consecutive HAMP domains. For visualization of their abundance and distribution (Figure 1b), a tree was constructed using GTDB taxonomy (Parks et al., 2020) for Bacteria and Archaea, and published classifications for Fungi (Spatafora et al., 2016) and other eukaryotes (Burki et al., 2020).

### 4.2 AlphaFold2 modeling

In total, 120 kinase‐ and 99 chemoreceptor‐associated poly‐HAMP arrays were selected for modeling with AlphaFold2 (Supplementary Table 2) both as full‐length arrays and as individual HAMP domains. Selection was based on sequence clustering of all identified HAMP domains using MMseqs2 (min_identity = 0.45, coverage = 0.5, cov_mode = 0, cluster_mode = 0) to obtain a diverse set; the corresponding full‐length arrays containing these representative HAMPs were then retrieved. Modeling was performed with AlphaFold2 (version 2.3.1) (Jumper et al., 2021) in AlphaFold‐Multimer mode (Evans et al., 2021) (dimers), 5 predictions per AF2 model (a total of 25 predictions), and without templates. The models were sorted by ranking confidence (0.8*ipTM + 0.2*pTM), and the top‐scoring (rank 1) model was chosen for subsequent analyses.

To compare modeling confidence between HAMP domains modeled in isolation and in arrays, per‐residue pLDDT scores for individual HAMP domains modeled in isolation and within arrays were extracted, averaged, and plotted as distributions (Figure 6, middle panel). These analyses were complemented by Rosetta 3.12 scoring (Leaver‐Fay et al., 2011), where models were subjected to fast relax with coordinate restraints and evaluated using averaged per‐residue Rosetta total energy scores computed with the ref2015 score function (Alford et al., 2017) (Figure 6, right panel).

### 4.3 Coiled‐coil parameters measurement

For quantifying conformations of isolated and arrayed HAMP domain models, we used Crick's equations as implemented in the SamCC Turbo tool (Szczepaniak et al., 2021). A key requirement of this analysis is a consistent coiled‐coil register assignment, which ensures that functionally corresponding regions are compared across analyzed HAMP domains regardless of their type. As described in the “Sequence analysis” section, each query MSA used to identify HAMP domains in the NR90 database was manually assigned a coiled‐coil register. These register annotations were propagated to all identified HAMP domains and used as a reference for measuring axial helix rotation of the input (N‐terminal) and output (C‐terminal) helices, as described in detail in our previous work (Winski et al., 2024).

### 4.4 Cloning, expression and purification

DNA fragments encoding AskA's N‐terminus together with the first four (residues 1–212, “4‐HAMP”) or the first six (residues 1–304, “6‐HAMP”) HAMP domains were obtained from *M. xanthus* DK1622 genomic DNA (Kaiser, 1979) by PCR, using primers adding an NdeI restriction site to the start and an extension comprising an Eco31I site, a sequence encoding a Gly‐Ser‐Gly linker and an XhoI restriction site to the end. The PCR products were ligated to the NdeI and XhoI sites of pET28b(+), thereby adding an N‐terminal His_6_ tag and a thrombin cleavage site. After treatment with XbaI and Eco31I, the excised fragments were cloned into pIBA‐GCN4di, a vector derived from pASK‐IBA2 (IBA Lifesciences GmbH) by N‐ and C‐terminal insertions of a codon‐optimized dimeric leucine zipper (GCN4 from *Saccharomyces cerevisiae*) while preserving its multiple cloning site (analogous to pIBA‐GCN4tri (Hernandez Alvarez et al., 2008)). In the final constructs, the N‐terminal His_6_‐Tag was retained and the C‐terminal 29 residue modified GCN4 sequence (MKQLEWKVEELLSKNYHLENEVARLKKLV) formed a continuous coiled coil in a register compatible with that of the last HAMP helix to which it was fused, including the GSG linker residues.

*Escherichia coli* C43 (DE3) cells (4‐HAMP) or Rosetta2 cells (6‐HAMP) were transformed with the constructs and cells were grown in LB medium supplemented with ampicillin. Protein expression was induced at an OD_600_ of 0.5–0.6 with anhydrotetracycline (0.25 μg/mL culture) at 25–28°C for 6–8 h. Cells were harvested by centrifugation. We found that the presence of urea was advantageous for protein solubility and stability (Dines et al., 2007). Thus, the cell pellets were resuspended in lysis buffer (50 mM Tris, 300 mM NaCl, 1 mM MgCl_2_, 2 M urea, 0.5 mM dithiothreitol (DTT), 10% glycerol, pH 8, supplemented with DNaseI, lysozyme and phenylmethyl sulfonylfluoride). Lysis was performed with a French Press. Cleared lysate was loaded onto an equilibrated 10 mL Ni‐NTA column. The column was washed with 10% elution buffer (as lysis buffer but containing 300 mM imidazole) and the protein was eluted with 100% elution buffer. Eluted protein was dialyzed against 20 mM Tris, 100 mM NaCl, 1.5 M urea, 1 mM MgCl_2_, 2 mM DTT, 5% glycerol, 10 mM imidazole, pH 7.8. White precipitate formed during dialysis, but the supernatant still contained sufficient amounts of the proteins which could be concentrated in 10 kDa spin concentrators. The purity of the isolated proteins was confirmed by SDS‐PAGE.

### 4.5 Crystallization, data collection, and structure solution

Crystallization was performed at 20°C via the vapor diffusion method. Initial trials were set up with a Honeybee 961 robot (Genomic Solutions Ltd.) by mixing 300–400 nL of protein solution and the same amount of reservoir solution on 96‐well sitting‐drop microplates, using commercially available screens (“Nextal,” Qiagen). Initial hit conditions were reproduced and refined in hanging‐drop setups using EasyXtal plates (Qiagen). Best‐diffracting crystals of 4‐HAMP were obtained with a protein concentration of 5.3 mg/mL in condition G11 of the Classic II Screen (25% PEG 3350, 0.1 M BisTris pH 6.5, 0.2 M MgCl_2_). For phasing, heavy‐atom derivatives of 4‐HAMP crystals were obtained by soaking with platinum salts. Prior to flash‐cooling, all 4‐HAMP crystals were cryoprotected with 15% glycerol. Best‐diffracting crystals of 6‐HAMP were found at a protein concentration of 17 mg/mL in condition H12 of the Classic I Screen (12% PEG 20000, 0.1 M MES pH 6.5). These crystals were sensitive to aging and had to be harvested immediately after their formation for good diffraction. Cryoprotection was done with 20% PEG 300 and 0.4 M urea.

All data for 4‐HAMP and 6‐HAMP crystals were collected on beamline X10SA at the Swiss Light Source at 100 K. Derivative data were collected on a MarCCD 225‐mm charge‐coupled device (CCD) detector at the Pt L‐III edge (peak); all native data were collected using a Pilatus 6M hybrid pixel detector (DECTRIS) at a wavelength of 1 Å. XDS (Kabsch, 2010) was used for processing and scaling of the diffraction data, with the statistics given in Supplementary Table 3. Phasing of 4‐HAMP crystals was done in two steps. First, initial phases were obtained following the Multiple Isomorphous Replacement with Anomalous Scattering protocol with one native and two diammino platinum dinitrite derivative datasets. SHELXD (Sheldrick, 2008) was employed for heavy atom location as implemented in autoSHARP (Vonrhein et al., 2007), followed by substructure refinement using SHARP (de La Fortelle & Bricogne, 1997). After density modification with Solomon (Abrahams & Leslie, 1996), the resulting map was still too noisy for manual or automated interpretation. For this reason, in a second step, we employed phased molecular replacement aided by the Solomon‐improved phases as implemented in MOLREP (Vagin & Teplyakov, 2010) with different HAMP domain models as search models. The first successful phased search was conducted with a two‐HAMP module derived from Aer2 (Airola, Watts, Bilwes, & Crane, 2010), followed by a successful phased search employing a single Af1503 HAMP domain (Ferris et al., 2011) as a search model, thereby locating the first three HAMP domains of the construct. After initial manual model building in Coot (Emsley & Cowtan, 2004) and refinement with REFMAC5 (Murshudov et al., 1999), a further molecular replacement search with MOLREP could locate the C‐terminal GCN4 adaptor using PDB entry 1GK6 (Strelkov et al., 2002) as a search model. The final structure comprises two chains in the ASU forming a single 4‐HAMP dimer. Phasing of 6‐HAMP crystals was performed by molecular replacement in MOLREP (Vagin & Teplyakov, 2010) using the 4‐HAMP structure as a search model, locating four chains in the ASU; these belong to three 6‐HAMP dimers, two of which are built by two‐fold crystallographic symmetry. All structures were completed by cyclic manual modeling with Coot (Emsley & Cowtan, 2004) and refinement with REFMAC5 (Murshudov et al., 1999) with local NCS restraints. In both structures, apart from the N‐terminal 25–30 residues, the linker in the last HAMP domains, and the C‐terminal portions of the GCN4 adaptors, the full chain of all poly‐HAMP molecules could be traced in the electron density. The refinement statistics are given in Supplementary Table 3; coordinates and structure factors were deposited in the PDB under accession codes 9TRJ (4‐HAMP) and 9TRK (6‐HAMP).

### 4.6 Molecular dynamics simulations

The simulations were performed in GROMACS 2024.3 (Abraham et al., 2015; Van Der Spoel et al., 2005) using the CHARMM36m force field (Huang et al., 2017). Bond vibrations were constrained with the LINCS algorithm (Hess, 2008). Long‐range electrostatics were treated with the PME method, and nonbonded interactions were computed using the Verlet cutoff scheme with a 1.2‐nm real‐space cutoff. A 2‐fs integration time step was used. Systems were solvated with TIP3P water in a dodecahedral periodic simulation box with a minimum solute–box distance of 1.4 nm, and the net charge was neutralized with Na^+^/Cl^−^ ions as needed. Energy minimization was performed using the steepest‐descent algorithm until the maximum force was below 300 kJ mol^−1^ nm^−1^. Following 1 ns equilibration in both the NVT and NPT ensembles, production simulations were run for 500 ns in triplicate. Temperature was controlled with the V‐rescale thermostat, and pressure was maintained at 1 bar using isotropic coupling with the C‐rescale barostat.

This initial set of simulations was carried out at 300 K using the arrayed and isolated AlphaFold2 models of the fifth HAMP domain of the CAA9318664.1 array, corresponding to residues 214–256. Helical rotation was measured using residues 217–227 and 244–254, corresponding to the N‐ and C‐terminal HAMP helices, respectively. During the simulations, all systems remained globally stable in terms of RMSD, but simulations started from the arrayed model showed rapid transition to the rotational state seen in the isolated model. To slow this convergence, a second set of simulations was performed at 250 K for 200 ns in triplicate. Even at reduced temperature, the arrayed HAMP rapidly adopted the x‐da state corresponding to the conformation observed in the isolated form (Figure 7). The PDB snapshots from the 250 K simulations are available through Figshare at https://doi.org/10.6084/m9.figshare.32230659.

## AUTHOR CONTRIBUTIONS

**Murray Coles:** Conceptualization; investigation; writing – original draft; writing – review and editing; formal analysis. **Stanislaw Dunin‐Horkawicz:** Conceptualization; investigation; writing – original draft; methodology; visualization; writing – review and editing; software; formal analysis; project administration; supervision; validation; data curation. **Reinhard Albrecht:** Investigation; validation; formal analysis; writing – original draft; writing – review and editing. **Marcus D. Hartmann:** Conceptualization; investigation; formal analysis; supervision; data curation; validation; writing – review and editing. **Jörg Martin:** Investigation; formal analysis. **Andrei N. Lupas:** Conceptualization; investigation; supervision; resources. **Carolin P. Ewers:** Conceptualization; investigation; formal analysis; validation. **Malgorzata Orlowska:** Software; formal analysis; writing – original draft. **Mikel Martinez‐Goikoetxea:** Writing – original draft; software; formal analysis; writing – review and editing.

## FUNDING INFORMATION

This work was supported by institutional funds from the Max Planck Society. S.D‐H. was additionally supported by the National Science Centre, Poland (grant no. 2020/37/B/NZ2/03268).

## CONFLICT OF INTEREST STATEMENT

The authors declare no conflicts of interest.

## Supporting information


**Supplementary Table 1.** Poly‐HAMP chemoreceptors and kinases whose HAMP domains were used as seed queries for searching the NCBI non‐redundant sequence database.
**Supplementary Table 2**. Poly‐HAMP chemoreceptor and kinase arrays modeled in AlphaFold2 either as full‐length arrays or as individual HAMP domains.
**Supplementary Table 3**. Crystallographic Data Collection and Refinement Statistics.
**Supplementary Figure 1**. Crystal packing of the 6‐HAMP construct. Top view from the N‐terminal side showing the four chains present in the asymmetric unit and the neighboring symmetry‐related copies required to complete the dimers. Chains A and B form a complete dimer within the asymmetric unit and are shown in red and green. Chains C and D, present as separate monomers in the asymmetric unit, are shown in blue and violet. The two neighboring symmetry‐related copies that complete their dimers are shown in two shades of gray.
**Supplementary Figure 2**. Close‐up of the non‐canonical‐to‐canonical interface, shown analogously to the canonical‐to‐non‐canonical interface in Figure 2c. For clarity, only the N‐terminal helix of the non‐canonical HAMP2 and the C‐terminal helix of the canonical HAMP3 are shown. In this interface, Trp is replaced by Phe155, and no corresponding side‐chain‐to‐backbone hydrogen bond is observed.
**Supplementary Figure 3**. Variability among ranked AlphaFold2 models. For each HAMP domain, the dRMSD of each rank 2–25 model relative to rank 1, calculated using Cα atoms of corresponding residues, was compared with the dRMSD between the corresponding isolated and array‐derived rank‐1 models, which served as the baseline. Bars show the fractions of rank 2–25 models below this baseline, above the baseline while retaining the expected compact parallel four‐helix‐bundle geometry, or above the baseline with aberrant HAMP geometry. Labels above the bars indicate the total fraction of models above the baseline. Aberrant geometries denote models that did not form the expected compact parallel four‐helix bundle, as assessed from the relative orientation of the four HAMP helix axes and the distances between helix centers.
**Supplementary Figure 4**. Distributions of conformational states, pLDDT scores, and Rosetta scores for arrayed (blue) and isolated (gray) HAMP domains from kinase‐associated arrays (the upper two rows) and chemoreceptor‐associated arrays (the lower two rows). In contrast to Figure 6 in the main text, canonical and non‐canonical HAMP domains are shown separately.
**Supplementary Figure 5**. dRMSD analysis of the MD trajectories shown in Figure 7. Trajectories from all three replicates are shown, with isolated and array‐derived simulations indicated in shades of blue and orange, respectively. dRMSD values were calculated from pairwise C*α* distance matrices relative to the isolated and array‐derived AlphaFold2 models used as the pre‐equilibration starting structures for the MD simulations and are plotted on the *x*‐ and *y*‐axes, respectively. Lines represent 10‐frame rolling means. Triangles indicate the first analyzed MD frames, whereas stars indicate the final plotted points.

## Data Availability

The data that support the findings of this study are available from the corresponding author upon reasonable request.
