# Supplementary material for: Conformational diversity in poly‐HAMP arrays and its implications for signal transduction
Source: Protein Sci. 2026 Aug 6;35(9):e70743. doi: 10.1002/pro.70743 (PMC13446029; doi:10.1002/pro.70743)
Supplement: Supplementary file 1 [file PRO-35-e70743-s001.docx]

**Supplementary Table 1.** Poly-HAMP chemoreceptors and kinases whose HAMP domains were used as seed queries for searching the NCBI non-redundant sequence database.

| Kinases | A0A1N6G1D1, A0A4R4P399, A0A248K2C0, I4YVW6, A0A542ZIX7, A0A1U7JAJ8, A0A4Z0BGY9, A0A1G9HBX6, A0A3E0HHV0, A0A1N7CPU7, A0A3A9YLK8, K9PBI3, G0G0R7, D8FVA6, A0A0X3XHH5, A0A1H5PQZ5, A0A2W2M3F7, A0A2U1VYS9, B0UB08, A0A2D3U389, A0A1Q9RWB5, A0A0X3RTC7, A0A2A3HZJ6 |
| --- | --- |
| Chemoreceptors | Q2FN62, Q2FQJ9, Q2FQT7, Q74DM2, B9M9P4, Q3BU77, Q3BU80, A0A1S8RTT3, Q2FRC3, Q5H2U8, B2FI15 |

**Supplementary Table 2.** Poly-HAMP chemoreceptor and kinase arrays modeled in AlphaFold2 either as full-length arrays or as individual HAMP domains.

| Kinases | ABF13477, ABI63596, ACK51402, AEW99355, AKF79476, AOR06392, AOW78957, APR78731, APV48351, BAU86971, CAA9213639, CAA9276420, CAA9318664, CAA9514814, CAA9541585, CAD6583708, CAF9929585, CAG8476750, CAG8501670, CAH2352000, CAI5760408, CAJ65078, CDH51117, CDH57830, CDS55145, CUI03596, EFL09696, GDY41841, GET40449, HAK93061, HBE57325, HJE60676, KAA0680100, KAF2719822, KAF7332700, KAF7747413, KAF8308501, KAF8737078, KAG0199653, KAG8912441, KAG8920499, KAH8922829, KAH8924750, KAI0338803, KAI9749930, KAJ1945458, KAJ1957453, KAJ2455678, KAJ3014897, KAJ7025691, KAJ7714292, KFN46324, KJA22434, MBA2310897, MBA2348676, MBA2404959, MBA2794146, MBA3245812, MBA3466498, MBA3561234, MBA3594958, MBA3664223, MBA3684214, MBA4156052, MBC7174319, MBC7448616, MBC7893908, MBE3086571, MBF6568894, MBI1912230, MBI2378142, MBI2386612, MBI3313561, MBI3359409, MBI3558156, MBI4205782, MBI4348258, MBI4811967, MBI5438214, MBI5607008, MBK9001788, MBK9201114, MBN9394845, MBT3386106, MBT9495849, MBV52702, MBV8298408, MBV9866075, MBW3637220, MBX3187116, MBX3198964, MBX6388644, MCC7539601, MCG8634810, MCH8347281, MCJ7470134, MCU0534516, MPY94410, NRQ38315, NUO48611, NUO51383, OLD49681, OLE64068, QFG26374, RKP25275, TFW05571, TMA17525, TMA20250, TWV11213, WP_013130450, WP_029722888, WP_051794678, WP_092264814, WP_136535146, WP_141958392, WP_147304648, WP_228057440, XP_003889300, XP_036637199, XP_047801733 |
| --- | --- |
| Chemoreceptors | CUU50852, ESS73188, GAK55060, HBC93786, MBF0361290, MBK7877023, MBK8338217, MBM2814453, MBN2695045, MBN2695612, MBN2833792, MBS4050147, MCB1190930, MCB9496075, MCD6207395, MCG2797269, MCH2340873, MCM2335051, MCP5495817, MCP5502499, MCT4632982, NLL09390, NLO19448, NOT67721, NOU23061, NOX88659, NRB67684, NUN71418, NVM22399, OPZ36790, OQA77434, PKL59521, PKL60757, PZM89608, TMN88776, TQD27560, WP_008429341, WP_012631780, WP_013484833, WP_019897594, WP_020565711, WP_041362574, WP_071392859, WP_091471401, WP_109941520, WP_109966919, WP_110186788, WP_124551638, WP_128694700, WP_135279084, WP_140003491, WP_148602150, WP_155468411, WP_157202402, WP_159355165, WP_162299629, WP_166404993, WP_190259213, WP_202905785, WP_207688227, WP_207688826, WP_209620254, WP_210996399, WP_211531235, WP_214421110, WP_221063350, WP_221064443, WP_226636625, WP_228519770, WP_229716082, WP_230838595, WP_235811085, WP_236210111, WP_238916629, WP_239441545, WP_248342755, WP_248343955, WP_248345650, WP_248345651, WP_248345920, WP_254458424, WP_255332674, WP_256601726, WP_256613233, WP_257743022, WP_257743469, WP_258346527, WP_260610421, WP_260788041, WP_263393292, WP_263634519, WP_269745533, WP_274396358, WP_274427672, WP_275355203, WP_275356878, WP_275357030, WP_275357036, WP_277275308 |

**Supplementary Table 3.** Crystallographic Data Collection and Refinement Statistics

| Structure | **4HAMP** | **6HAMP** |
| --- | --- | --- |
| **Data collection** |  |  |
| Space group | P2_1_2_1_2_1_ | P2_1_2_1_2 |
| Cell parameters  a, b, c, (*Å)* | 39.17, 69.24, 181.45 | 59.76, 105.21, 194.84 |
| Wavelength (*Å)* | 1.000 | 1.000 |
| Resolution limits (*Å)^a^* | 38.3 – 2.05 (2.17 – 2.05) | 39.0 – 1.85 (1.96 – 1.85) |
| Unique reflections | 31524 (4951) | 103844 (15707) |
| Completeness (%) | 98.7 (97.8) | 98.1 (93.1) |
| Redundancy | 3.60 (3.60) | 3.58 (3.26) |
| I/σI | 9.75 (1.81) | 11.16 (1.66) |
| R_merge_ (%) | 9.0 (72.5) | 8.0 (60.6) |
| CC (1/2) | 99.7 (80.3) | 99.9 (78.7) |
| **Refinement** |  |  |
| Resolution limits (*Å)* | 38.3 – 2.05 | 39.0 – 1.85 |
| R_cryst_ (%) | 23.6 | 20.1 |
| R_free_ (%) | 27.1 | 24.4 |
| Mean B value (*Å^2^)* | 46.1 | 30.7 |
| Ramachandran preferred/allowed/outlier regions (%) | 99.0/1.0/0.0 | 99.6/0.4/0.0 |
| **PDB accession code** | **9TRJ** | **9TRK** |

^a^ Values in parenthesis refer to the highest-resolution shell.

**Supplementary Figure 1.** Crystal packing of the 6-HAMP construct. Top view from the N-terminal side showing the four chains present in the asymmetric unit and the neighboring symmetry-related copies required to complete the dimers. Chains A and B form a complete dimer within the asymmetric unit and are shown in red and green. Chains C and D, present as separate monomers in the asymmetric unit, are shown in blue and violet. The two neighboring symmetry-related copies that complete their dimers are shown in two shades of gray.

**Supplementary Figure 2. Close-up of the non-canonical-to-canonical interface**, shown analogously to the canonical-to-non-canonical interface in Figure 2C. For clarity, only the N-terminal helix of the non-canonical HAMP2 and the C-terminal helix of the canonical HAMP3 are shown. In this interface, Trp is replaced by Phe155, and no corresponding side-chain-to-backbone hydrogen bond is observed.

**Supplementary Figure 3. Variability among ranked AlphaFold2 models.** For each HAMP domain, the dRMSD of each rank 2–25 model relative to rank 1, calculated using Cα atoms of corresponding residues, was compared with the dRMSD between the corresponding isolated and array-derived rank-1 models, which served as the baseline. Bars show the fractions of rank 2–25 models below this baseline, above the baseline while retaining the expected compact parallel four-helix-bundle geometry, or above the baseline with aberrant HAMP geometry. Labels above the bars indicate the total fraction of models above the baseline. Aberrant geometries denote models that did not form the expected compact parallel four-helix bundle, as assessed from the relative orientation of the four HAMP helix axes and the distances between helix centers.

**Supplementary Figure 4.** Distributions of conformational states, pLDDT scores, and Rosetta scores for arrayed (blue) and isolated (grey) HAMP domains from kinase-associated arrays (the upper two rows) and chemoreceptor-associated arrays (the lower two rows). In contrast to Figure 6 in the main text, canonical and non-canonical HAMP domains are shown separately.

**Supplementary Figure 5. dRMSD analysis of the MD trajectories shown in Figure 7.** Trajectories from all three replicates are shown, with isolated and array-derived simulations indicated in shades of blue and orange, respectively. dRMSD values were calculated from pairwise Cα distance matrices relative to the isolated and array-derived AlphaFold2 models used as the pre-equilibration starting structures for the MD simulations and are plotted on the x- and y-axes, respectively. Lines represent 10-frame rolling means. Triangles indicate the first analyzed MD frames, whereas stars indicate the final plotted points.
